# Oxidative Stress Inactivates Cobalamin-Independent Methionine Synthase (MetE) in Escherichia coli

**DOI:** 10.1371/journal.pbio.0020336

**Published:** 2004-10-05

**Authors:** Elise R Hondorp, Rowena G Matthews

**Affiliations:** **1**Department of Biological Chemistry, University of MichiganAnn Arbor, MichiganUnited States of America; **2**Biophysics Research Division and Life Sciences Institute, University of MichiganAnn Arbor, MichiganUnited States of America

## Abstract

In nature, Escherichia coli are exposed to harsh and non-ideal growth environments—nutrients may be limiting, and cells are often challenged by oxidative stress. For E. coli cells confronting these realities, there appears to be a link between oxidative stress, methionine availability, and the enzyme that catalyzes the final step of methionine biosynthesis, cobalamin-independent methionine synthase (MetE). We found that E. coli cells subjected to transient oxidative stress during growth in minimal medium develop a methionine auxotrophy, which can be traced to an effect on MetE. Further experiments demonstrated that the purified enzyme is inactivated by oxidized glutathione (GSSG) at a rate that correlates with protein oxidation. The unique site of oxidation was identified by selectively cleaving N-terminally to each reduced cysteine and analyzing the results by liquid chromatography mass spectrometry. Stoichiometric glutathionylation of MetE by GSSG occurs at cysteine 645, which is strategically located at the entrance to the active site. Direct evidence of MetE oxidation in vivo was obtained from thiol-trapping experiments in two different E. coli strains that contain highly oxidizing cytoplasmic environments. Moreover, MetE is completely oxidized in wild-type E. coli treated with the thiol-oxidizing agent diamide; reduced enzyme reappears just prior to the cells resuming normal growth. We argue that for E. coli experiencing oxidizing conditions in minimal medium, MetE is readily inactivated, resulting in cellular methionine limitation. Glutathionylation of the protein provides a strategy to modulate in vivo activity of the enzyme while protecting the active site from further damage, in an easily reversible manner. While glutathionylation of proteins is a fairly common mode of redox regulation in eukaryotes, very few proteins in E. coli are known to be modified in this manner. Our results are complementary to the independent findings of Leichert and Jakob presented in the accompanying paper (Leichert and Jakob 2004), which provide evidence that MetE is one of the proteins in E. coli most susceptible to oxidation. In eukaryotes, glutathionylation of key proteins involved in protein synthesis leads to inhibition of translation. Our studies suggest a simpler mechanism is employed by E. coli to achieve the same effect.

## Abstract 2

Under conditions of oxidative stress, methionine auxotrophy in *E. coli* is a result of glutathionylation of the enzyme that catalyzes the final step of methionine biosynthesis (MetE)

## Introduction

As a consequence of living in an aerobic world, organisms face the challenge of maintaining a favorable cellular redox status. Oxidative stress is caused by imbalances between the production and disposal of reactive oxygen species, which can damage proteins, lipids, and nucleic acids. Bacteria encounter reactive oxygen intermediates that are generated as byproducts of aerobic metabolism or during challenge by the immune systems of hosts. Thus, understanding the effects of oxidative stress on the cell, as well as elucidating cellular defense mechanisms, is of considerable interest.

**Figure 1:**
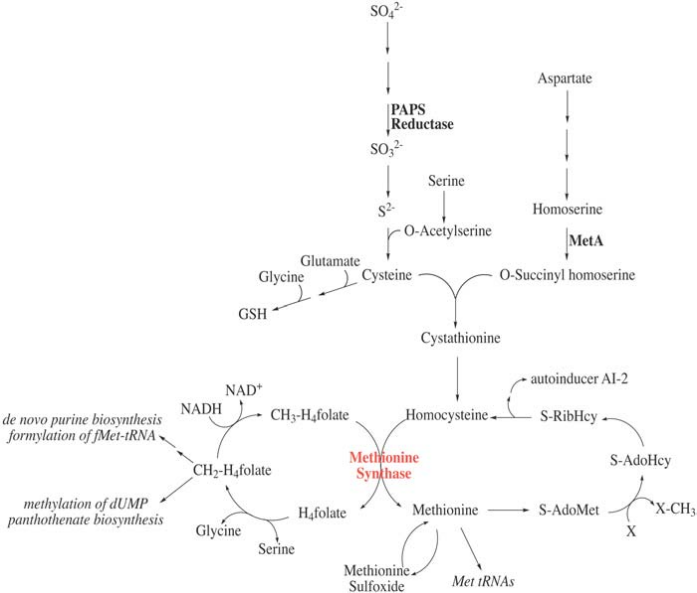
Pathways of Methionine Biosynthesis and One-Carbon Metabolism in E. coli

Over the past several decades, multiple observations in Escherichia coli have been reported that collectively suggest a link between oxidative stress, methionine limitation, and the enzyme that catalyzes the final step in methionine biosynthesis, cobalamin-independent methionine synthase (MetE). In *E. coli,* the sulfur of methionine comes from cysteine, which in turn obtains the sulfur group from sulfate via the sulfate assimilation pathway (Figure 1). In catalyzing the formation of methionine, MetE lies at the intersection between the methyl cycle and the one-carbon pathway. Several studies involving cellular adaptations to stress conditions have reported unusual expression of MetE under stress conditions. Following the transition from anaerobic to aerobic growth in minimal medium, wild-type E. coli cells were found to rapidly induce MetE to a level comprising almost 5% of the total cellular protein (Smith and Neidhardt 1983). Similarly, MetE was massively overexpressed in a temperature-sensitive mutant of *groEL*. GroEL is a heat-shock protein that is instrumental in helping to correctly fold cellular proteins. When the *groEL* strain growing in rich medium was shifted to the nonpermissive temperature, MetE was the major soluble protein synthesized (Horwich et al. 1993). The additional inability of this strain to grow on minimal medium suggests that MetE may not be correctly functioning in these cells. Furthermore, Candida albicans MetE has been found to be heat and estrogen inducible, and is believed to play an important role in the yeast stress response and virulence (Burt et al. 1999). The basis for the overproduction of MetE in all of these experiments has been unclear.

In addition, several independent researchers have reported an association between stress conditions and methionine limitation in E. coli. Early studies found that strains lacking the genes coding for the manganese and iron superoxide dismutases (*sodA* and *sodB,* respectively) were sensitive to oxidative challenge and had an oxygen-dependent growth requirement for the sulfur-containing amino acids cysteine and methionine (Carlioz and Touati 1986; Benov et al. 1996), which was attributed to leakage of sulfite from the cell. In *trxA grxA* (thioredoxin 1 and glutaredoxin 1) and *grxA grxB grxC gor* (glutaredoxins 1, 2, and 3 and glutathione reductase) strains of *E. coli,* cells required cysteine or methionine for growth, presumably because of oxidative inactivation of 3′-phosphoadenylylsulfate (PAPS) reductase (Russel et al. 1990; Vlamis-Gardikas et al. 2002; Lillig et al. 2003). Similarly, the growth of E. coli was dramatically slowed following a shift to higher temperature, unless the medium was supplemented with methionine (Ron and Davis 1971). This heat-induced methionine auxotrophy was traced to temperature sensitivity of homoserine transsuccinylase (MetA), the enzyme that catalyzes the first step in methionine biosynthesis (Ron and Davis 1971; Ron and Shani 1971; Gur et al. 2002); however, MetE has also been subsequently identified as a thermolabile protein in heat-treated E. coli (Mogk et al. 1999). It is intriguing that MetE was a major aggregation-prone protein at a temperature (45 °C) well below the melting temperature of the purified protein (55 °C) (E. Hondorp and R. Matthews, unpublished data). Aerobic heat shock appears to be associated with oxidative stress (Benov and Fridovich 1995), suggesting that the aggregation of MetE might relate to oxidative stress, rather than heat per se. Though oxidative stress undoubtedly has multiple effects, taken together, these observations suggest that MetE may be inactivated by biological oxidants, thereby decreasing the production of methionine within the cell.

Methionine biosynthesis involves several enzymes (Figure 1); the final step is catalyzed by either cobalamin (B_12_)-independent methionine synthase (the *metE* gene product) or B_12_-dependent methionine synthase (the *metH* gene product). Both enzymes employ a catalytic zinc to transfer a methyl group from methyltetrahydrofolate to L-homocysteine to form methionine (equation 1).



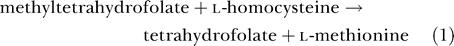



While mammals have only the MetH homolog, organisms that do not synthesize or transport vitamin B_12_ (e.g., yeast, fungi, and plants) use only the B_12_-independent isoform (Matthews 1984). The E. coli genome contains both *metE* and *metH* but they are differentially expressed: functional MetH is only expressed in the presence of B_12_, which also serves to repress MetE expression. In the absence of exogenously supplied B_12_, MetE is the sole source of de novo methionine synthesis (Greene 1996).

In this study, we investigated the effects of oxidative stress on MetE and methionine availability within the cell. Transient oxidative stress conditions induced by diamide were found to elicit a methionine auxotrophy in E. coli growing in minimal medium, which was traced to an effect on MetE. In vitro experiments demonstrated that oxidized glutathione (GSSG) reversibly oxidizes MetE at a rate that correlates with enzyme inactivation. GSSG was found to specifically glutathionylate MetE and induce a conformational change. In vivo thiol-trapping experiments provide direct evidence that MetE is readily oxidized in cells experiencing a variety of oxidative stress conditions. Moreover, oxidation of MetE is associated with a cellular dependence upon methionine for growth, suggesting that oxidant-mediated inactivation of MetE may have a profound impact on the stressed cell.

## Results

### Methionine Becomes Limiting for Growth in Cells Subjected to Oxidative Stress

**Figure 2:**
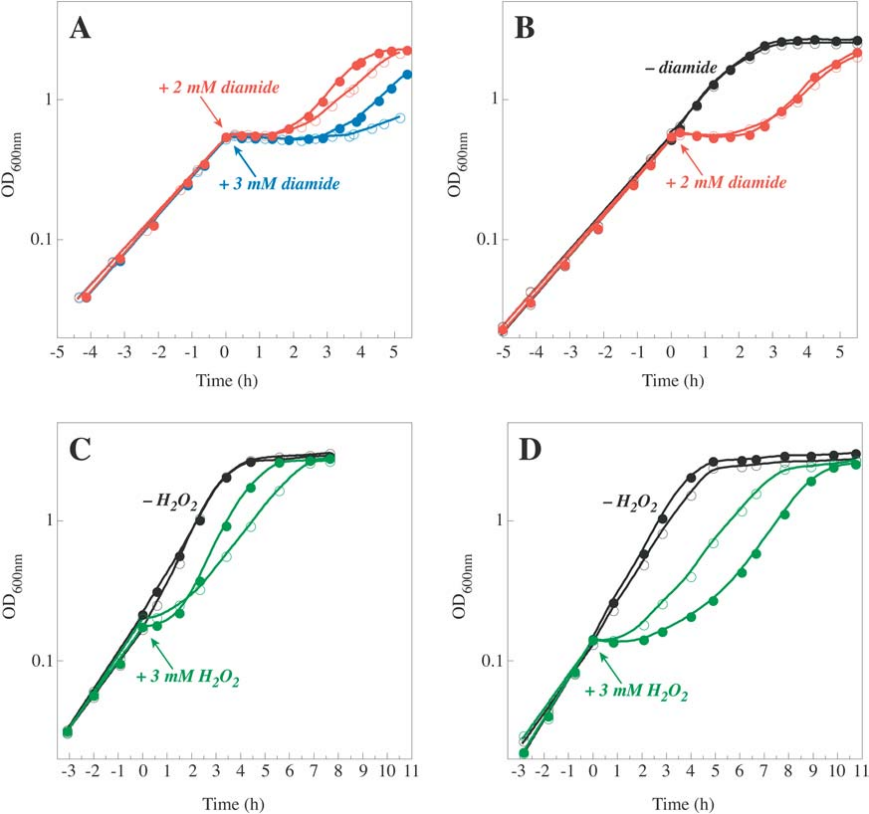
Methionine Limitation during Oxidative Stress Induced by Diamide and H_2_O_2_ (A) Cells of wild-type E. coli strain W3110 growing exponentially in glucose-minimal medium were diluted into the same medium with (filled circles) or without (open circles) 0.2 mM L-methionine. Cultures were allowed to grow to an OD_600_ of approximately 0.5, and then diamide was added to a final concentration of 2 mM (red) or 3 mM (blue), and growth was monitored. (B) Cultures were grown as in (A), except that they were diluted into medium with (filled circles) or without (open circles) 0.2 mM L-homocysteine. At an OD_600_ of approximately 0.5, diamide (final concentration, 2 mM) was added to one set of cultures (red). (C) Cells of wild-type E. coli strain W3110 were grown in the presence or absence of L-methionine as described in (A). At an OD_600_ of approximately 0.2, H_2_O_2_ was added to one set of cultures (green) to a final concentration of 3 mM (a concentration found to be high enough to induce a significant slowing of growth). (D) Cultures were grown as in (C) except that they were diluted into medium with (filled circles) or without (open circles) 0.2 mM L-homocysteine. Note the different time scales between (A,B) and (C,D).

Previous observations have suggested a link between oxidative stress and a growth requirement for methionine in E. coli. We investigated the duration of growth lags induced by oxidative stress in cells growing in glucose-minimal medium containing or lacking methionine. In the experiment shown in Figure 2A and 2B, oxidative stress was induced by addition of the thiol oxidant diamide, which rapidly penetrates cells and oxidizes intracellular thiols. Diamide treatment does not kill cells, but results in an abrupt arrest of growth for a length of time that is dependent on the initial concentration of diamide. Cells resume normal growth once the thiol status is restored (Kosower and Kosower 1995). E. coli cultures logarithmically growing in glucose-minimal medium with and without methionine experienced a lag in growth upon challenge with diamide (Figure 2A). However, cells grown in medium containing methionine resumed growth significantly faster than those needing to synthesize methionine. Higher diamide concentrations increased the duration of the growth lag and augmented the effect of methionine on resumption of growth.

The biosynthesis of methionine involves several reactions (see Figure 1). If a step upstream of the reaction catalyzed by MetE were responsible for methionine limitation during diamide treatment, addition of homocysteine to the medium should decrease the growth lag imposed by diamide. No effect of homocysteine addition was seen on the duration of the growth lag in diamide-treated cells (Figure 2B), even though homocysteine can support the growth of cells that lack enzymes upstream of MetE in the biosynthetic pathway (Urbanowski and Stauffer 1989).

Similar results were obtained when hydrogen peroxide (H_2_O_2_) was used to induce oxidative stress. Addition of methionine to the medium significantly decreased the growth lag produced by H_2_O_2_ treatment (Figure 2C), while homocysteine was ineffective and even amplified the lag (Figure 2D). The mechanism by which homocysteine appears to have potentiated the H_2_O_2_ stress is not known; however, it was clearly not protective. Taken together, the growth experiments suggest that methionine becomes limiting under oxidative stress, and that the bottleneck to de novo biosynthesis of methionine lies in the terminal step catalyzed by MetE.

### Inactivation of MetE In Vitro Is Correlated with Cysteine Oxidation

**Figure 3:**
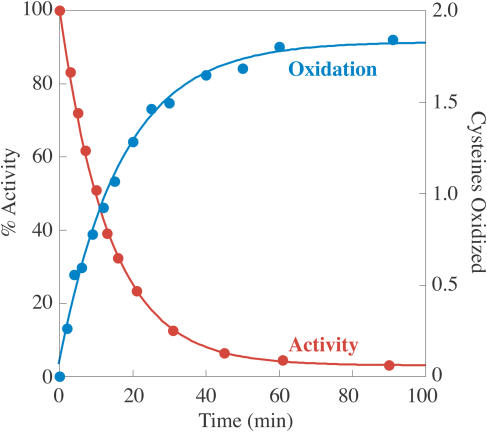
In Vitro Treatment of MetE with GSSG Leads to Loss of Activity and Cysteine Oxidation At time zero, GSSG (final concentration, 5 mM) was added to 50 μM MetE in 100 mM Tris chloride (pH 7.2). MetE activity was measured in samples removed at the indicated time points as described in the Materials and Methods. These assays are made over a 30-s time period and initiated by addition of MetE, so that oxidation of MetE by GSSG is minimal during the time required for measurement. Also, at the indicated times, an aliquot of the protein was precipitated with TCA, and the extent of cysteine oxidation was determined by DTNB titration. MetE activity loss (red) occurred with a rate constant of 0.07 min^−1^, while cysteine oxidation (blue) occurred with a rate constant of 0.06 min^−1^.

The methionine auxotrophy observed under oxidative stress conditions could reflect an increased demand for methionine or a decreased rate of de novo synthesis due to inactivation of MetE. To distinguish between these possibilities, we assayed the activity of the purified enzyme upon challenge with GSSG (Figure 3). Nearly complete loss of methionine synthase activity was observed following addition of GSSG. This process was readily reversible upon incubation with a reductant such as dithiothreitol (DTT) or reduced glutathione (GSH) (data not shown). Activity loss was correlated with thiol oxidation, as assessed by dithio-1,4-nitrobenzoic acid (DTNB) titration of the trichloroacetic acid (TCA)–precipitated protein. GSSG treatment led to thiol oxidation, which proceeded with approximately the same rate constant (0.06 min^−1^) as loss of activity (0.07 min^−1^).

While the rate of thiol oxidation could be measured with DTNB, the absolute number of cysteines oxidized was ambiguous. MetE contains seven cysteines, yet DTNB titration of the fully reduced enzyme gave values between 7.2 and 9.0 cysteines for different protein preparations, and oxidation of MetE led to an average decrease of 1.8 cysteines per mol. Thus it was important to use independent means to determine the number of cysteines oxidized by GSSG.

Oxidation of MetE by GSSG would be expected to proceed by initial formation of a mixed disulfide between MetE and glutathione (equation 2).



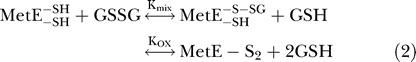



An inter- or intramolecular disulfide bond could then be generated by attack on the mixed disulfide by a second cysteine residue. These two possibilities can be distinguished by using mass spectrometry to determine the molecular mass of the oxidized protein. The reduced protein was found to be 84,530 ± 6 Da (expected mass, 84,542, −0.01% deviation), whereas the mass of the protein oxidized by GSSG was 84,835 ± 8 Da. The mass difference of 305 ± 10 Da between oxidized and reduced MetE is consistent with the addition of a single glutathione adduct (expected mass, 306 Da). Formation of an intramolecular disulfide bond would only decrease the mass by 2 Da, which would not be detected by mass spectrometry in such a large protein.

To obtain evidence for stoichiometric glutathionylation of MetE, the oxidized protein was reduced with DTT to release the glutathione adduct. The DTT and GSH were separated from the protein using a Microcon concentrator. The filtrate was assayed for GSH using fluorescamine, which reacts with primary amines to form a fluorescent product. The reduced protein did not release GSH (0.0 mol GSH per mol protein) while oxidized protein released 0.9 mol GSH per mol of protein. Thus both mass spectrometry and the fluorescamine assay were consistent with glutathionylation of MetE by GSSG.

### MetE Is Glutathionylated at Cysteine 645

**Figure 4:**
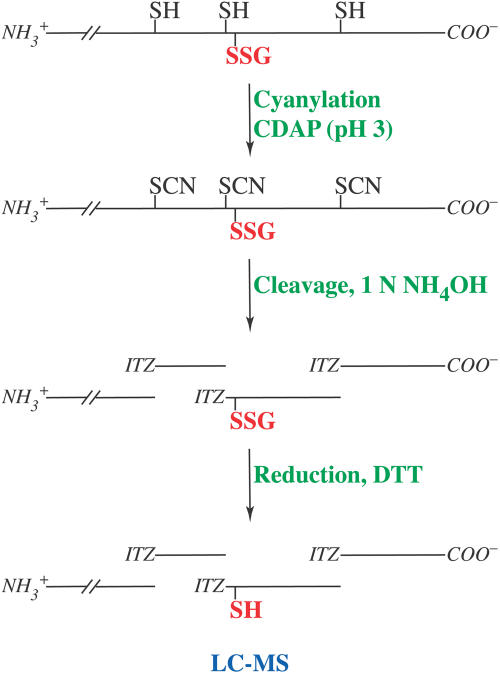
Strategy for Mapping of Disulfide Bonds Formed during Oxidative Inactivation of MetE In Vitro Protein sulfhydryls are cyanylated with CDAP under denaturing, acidic conditions, and then cleaved N-terminally to each cyanylated cysteine with 1 N ammonia. This treatment results in an N-terminal fragment and a series of itz peptides. The mixture is then completely reduced with DTT and analyzed by LC-MS. Cleavage does not occur adjacent to cysteines that are oxidized. Thus sites of oxidation can be deduced from the mass fingerprint.

In order to determine the site of oxidation and verify that an intramolecular disulfide was not being formed concomitant with oxidation, we performed disulfide mapping experiments based on the method of Wu and Watson (1997). MetE was cyanylated with 1-cyano-4-dimethylamino-pyridinium tetrafluoroborate (CDAP) under denaturing conditions at pH 3, and then cleaved immediately N-terminally to the cyanylated cysteines in 1 N ammonia, resulting in the formation of 2-iminothiazolidine-4-carboxyl (itz) peptides (Figure 4). Cleavage will not occur at cysteines that have been protected from cyanylation by oxidation. Following cleavage, any disulfide bonds were reduced with DTT and the resulting peptide fingerprint analyzed by liquid chromatography mass spectrometry (LC-MS).

This approach allows for relatively straightforward mapping of disulfide bonds. The acidic pH employed minimizes the possibility of disulfide bond scrambling. Furthermore, the ability to cleave N-terminally to each cyanylated cysteine allows cleavage between closely spaced, even adjacent, cysteines. These factors provide a significant advantage over traditional methods of peptide mapping, particularly for MetE, which contains cysteines spaced only two residues apart at the active site (cysteines 643 and 645). Moreover, for large proteins, most proteolytic and chemical cleavage methods have the potential to generate numerous peptides, whereas this approach produces a more limited number.

**Figure 5:**
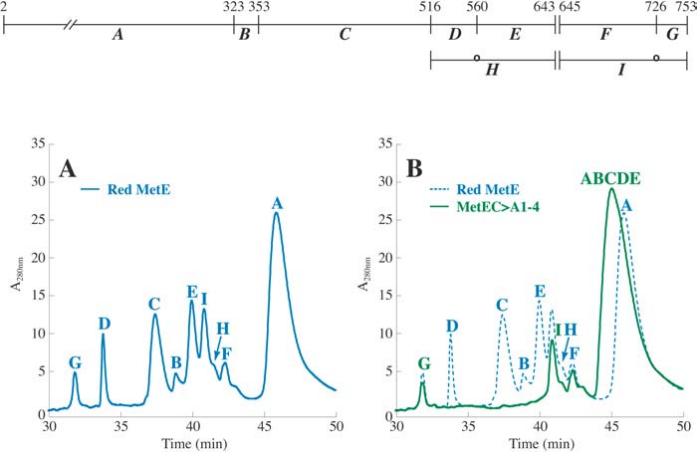
Disulfide Mapping of Reduced MetE Reduced MetE should be cleaved N-terminally to each of its seven cysteines to generate eight protein fragments as shown in the diagram, where peptide *A* comprises residues 2–322, *B* is itz323–352, *C* is itz353–515, *D* is itz516–559, *E* is itz560–642, *F* is itz645–725, and *G* is itz726–753. It is assumed that itz643–644 is too small to be resolved. β-elimination of cyanylated cysteines can also occur; *H* is itz516–642 with β-elimination at cysteine 560, and *I* is itz645–753 with β-elimination at cysteine 726. (A) The HPLC trace at 280 nm for disulfide mapping of fragments derived from cyanylation and cleavage of reduced MetE. Following derivitization, the samples were chromatographed on a C4 reversed-phase column as described in the Materials and Methods. Peaks were assigned from comparison of masses determined by mass spectrometry and predicted masses (see Table 1). (B) The HPLC trace for disulfide mapping of MetEC>A1–4 (green), which only contains cysteines 643, 645, and 726, overlaid with that of the wild-type reduced protein (blue). Peaks corresponding to *ABCDE*, *G*, and *I* were identified from the mass data (see Table 1). The mass for one peak could not be determined, but was assigned as *F* by comparison to the wild-type trace.

LC-MS analysis of the cyanylated and cleaved reduced MetE allowed for identification of all but one of the expected peptides (Figure 5A; Table 1). High performance liquid chromatography (HPLC) peaks with masses corresponding to each of the expected peptides were observed, except for the dipeptide itz643–644, which is too small to be detected. Cyanylated cysteines can also undergo a β-elimination side reaction instead of cleavage. Susceptibility of cyanylated cysteines to β-elimination depends on the neighboring residues (Wu and Watson 1998). In MetE, cysteine 726 readily formed the β-elimination product *I,* and β-elimination at cysteine 560 produced a small amount of product *H*. Identification of these side products provides additional evidence that cysteines 560 and 726 were reduced and available for cyanylation. Disulfide mapping of a mutant MetE, MetEC>A1–4, in which cysteines 323, 353, 516, and 560 were changed to alanines, further confirmed the mass assignments. Only peaks corresponding to cleavage next to the three remaining cysteines (643, 645, and 726) were observed (Figure 5B; Table 1).

**Table 1:**
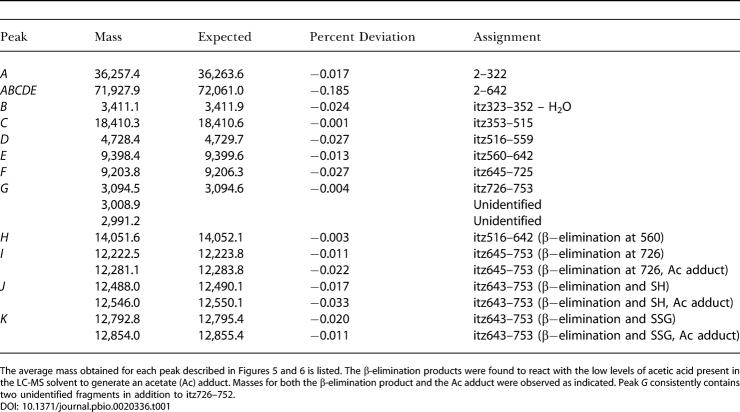
Mass Spectrometry Data for Disulfide Mapping Experiments

**Figure 6:**
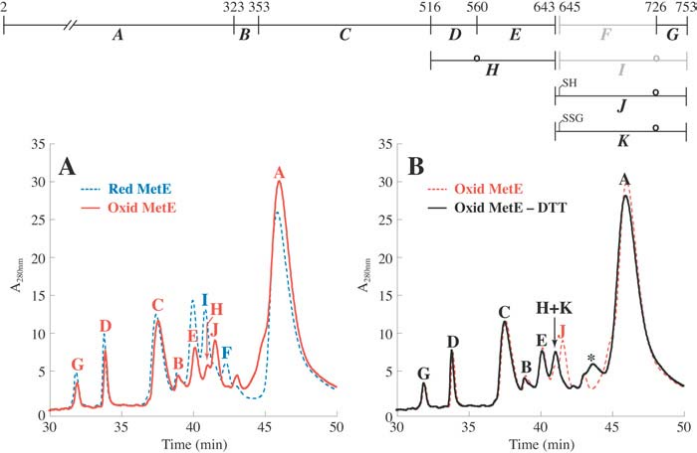
Disulfide Mapping of Oxidized MetE Reveals That Cysteine 645 Is Glutathionylated (A) LC-MS analysis of fragments generated by cyanylation, cleavage, and total reduction (see Figure 4) of GSSG-oxidized (red) and reduced (blue) MetE. For oxidized MetE, peaks corresponding to *F* and *I* were not found. Instead, a peak was observed with a mass corresponding to *J,* itz643–753, with one reduced uncyanylated cysteine and β-elimination at the other cysteine. As discussed in the text, β-elimination occurs preferentially at cysteine 726 in the reduced enzyme, and we infer that the reduced uncyanylated cysteine is cysteine 645. (B) LC-MS analysis of the cleavage products in (A) prior to DTT reduction (black) or following total reduction (red). Prior to DTT reduction, a peak is observed that has a mass corresponding to that expected for *K,* itz643–753, with a glutathione adduct at one cysteine and β-elimination at the other cysteine. As discussed in the text, β-elimination occurs preferentially at cysteine 726 in the reduced enzyme, and we infer that the glutathionylated cysteine is cysteine 645. The peak containing fragment *J,* the product of DTT reduction, is no longer apparent. The broad peak, indicated by the asterisk, appeared to contain a mixture of fragments with masses that could not be deconvoluted. These were attributed to disulfide-linked byproducts formed under the basic cleavage conditions prior to reduction with DTT.

The average mass obtained for each peak described in Figures 5 and 6 is listed. The β-elimination products were found to react with the low levels of acetic acid present in the LC-MS solvent to generate an acetate (Ac) adduct. Masses for both the β-elimination product and the Ac adduct were observed as indicated. Peak *G* consistently contains two unidentified fragments in addition to itz726–752

Disulfide mapping data for the oxidized protein following cyanylation, cleavage, and reduction with DTT (Figure 6A; Table 1), were similar to results for the reduced protein, suggesting that most of the cysteines in MetE remained reduced. Peptides *A, B, C, D, E,* and *H* provide evidence that cysteines 323, 353, 516, 560, and 643 were reduced. Peptides *G* and *J* indicate that cysteine 726 was reduced. The pattern is consistent with the initial oxidation occurring at cysteine 645 alone. Peptide *J* is the expected product obtained when MetE is initially oxidized at cysteine 645, with β-elimination at cysteine 726, and is then reduced.

LC-MS analysis of the oxidized protein fragments prior to DTT reduction yielded an almost identical pattern to that obtained after reduction (Figure 6B; Table 1). However, a peptide, *K,* corresponding to itz643–753 with a glutathione adduct and β-elimination, was observed instead of *J*. It is not possible to distinguish which cysteine, 645 or 726, is glutathionylated and which has undergone β-elimination based simply on the masses of peptides *J* and *K*. However, the presence of peptide *G* in all traces at a level similar to that of reduced MetE indicates that cysteine 726 must be reduced. Moreover, the high susceptibility of cysteine 726 to undergo β-elimination (as evidenced by peak *I* in Figure 5A)—contrasted with the complete lack of a β-elimination product at cysteine 645—argues that β-elimination occurs at cysteine 726 in the oxidized enzyme and glutathionylation occurs at cysteine 645.

**Figure 7:**
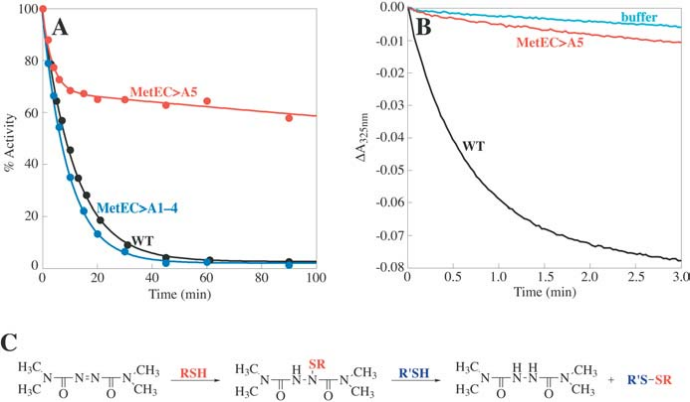
Cysteine 645 Is Critical for Sensitivity of MetE to Oxidation (A) The activity of 50 μM wild-type MetE (black), MetEC>A1–4 (blue), and MetEC>A5 (red) was assayed following addition of GSSG to a final concentration of 5 mM. 100% activity refers to the specific activity of the fully reduced enzyme prior to GSSG addition (27 min^−1^ for wild-type, 24 min^−1^ for mutant). (B) The change in absorbance at 325 nm was used to monitor diamide reduction after addition of 500 μM diamide to buffer (light blue), 50 μM wild-type MetE (black), and 50 μM MetEC>A5 (red). (C) Scheme for the reaction of MetE with diamide. In the first step, attack of cysteine 645 on diamide forms an inactive but stable complex. In vivo*,* where GSH is present in high concentration, attack of GSH leads to the formation of MetE-S-SG and reduced diamide.

In order to confirm that cysteine 645 is the only site of oxidation, the inactivation of various MetE mutants was analyzed. MetEC>A1–4 contains only cysteine 645 and the zinc-binding ligands, cysteines 643 and 726; the latter ligands are required for catalytic activity. This mutant has approximately 90% of the activity of the native protein, and the kinetics of inactivation by GSSG are nearly identical to that of the wild-type protein (Figure 7A). However, mutation of cysteine 645 to alanine (MetEC>A5) dramatically altered the course of inactivation by GSSG. The slow partial loss of activity seen with MetEC>A5 may reflect weakening of zinc binding for the mutant enzyme, allowing glutathione to compete with MetE for zinc.

Further evidence implicating cysteine 645 as the site of sensitivity to oxidation was obtained by monitoring the oxidation of MetE by diamide. The reaction of diamide with thiols can be followed at 325 nm, where diamide absorbs strongly, but the reduced product does not (Kosower and Kosower 1995). Addition of MetE to diamide results in a rapid decrease in absorbance at 325 nm (Figure 7B). However, MetEC>A5 does not appear to be oxidized by diamide. This observation suggests that diamide oxidation of MetE in vitro involves an initial attack of cysteine 645 on diamide to form an inactive covalent complex (Figure 7C). In the presence of accessible thiols, such as the millimolar concentrations of GSH present in vivo, this complex can then readily react to form glutathionylated MetE and reduced diamide. Thus, although diamide oxidation does not result in glutathionylation of MetE in the absence of glutathione, this experiment demonstrates the extreme reactivity of cysteine 645 to oxidizing agents.

### Glutathionylation of MetE Induces a Conformational Change

**Figure 8:**
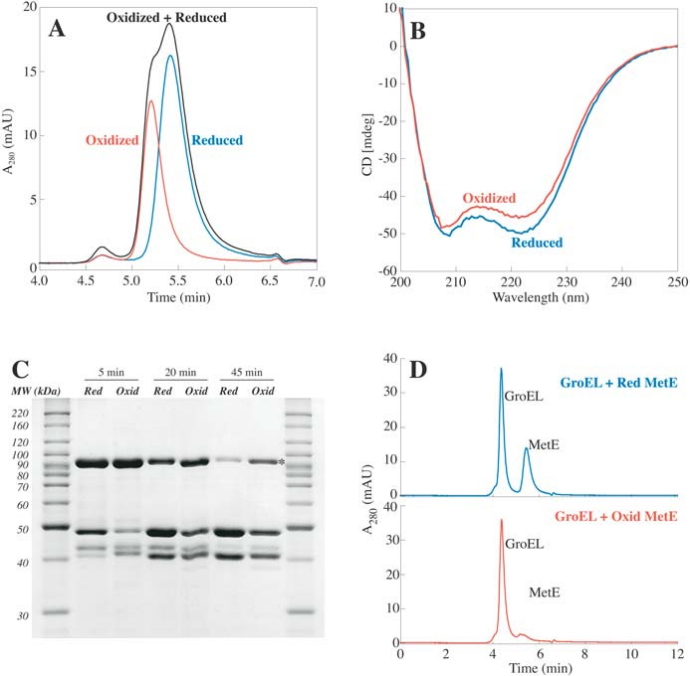
Glutathionylation of MetE Induces a Conformational Change (A) Gel filtration analysis of reduced (blue), GSSG-oxidized (red), or a mixture of reduced and GSSG-oxidized (black) MetE. (B) Far UV CD spectra of reduced (blue) and GSSG-oxidized (red) MetE. (C) SDS-PAGE analysis of a digestion of native MetE with 0.08% trypsin. The band corresponding to the molecular weight of the holo-protein is marked with an asterisk. (D) GroEL (2 μM) was incubated with 1 μM reduced (blue) or GSSG-oxidized (red) MetE and the proteins were then subjected to gel filtration.

In order to further characterize the effects of GSSG oxidation on MetE, the reduced and oxidized forms of the protein were subjected to gel filtration. The elution of glutathionylated MetE was markedly shifted in comparison to that for the reduced protein (Figure 8A). The change in hydrodynamic radius seen upon oxidation suggested that GSSG oxidation had altered the conformation of MetE. Therefore, to confirm a conformational difference between reduced and oxidized MetE, we analyzed the two forms of the protein by circular dichroism (CD) spectroscopy and limited tryptic digestion. The similarity between the CD spectra of oxidized and reduced MetE suggests that the oxidized protein has not undergone a gross structural rearrangement and is still largely intact (Figure 8B). However, the deviation in the two spectra between 210 and 230 nm is consistent with a conformational change upon glutathionylation. Tryptic digestion of oxidized MetE appeared to occur with different kinetics compared to that of the reduced protein (Figure 8C). The oxidized protein was more stable against tryptic digestion than the reduced form, again consistent with a conformational change upon MetE oxidation.

MetE has previously been shown to be an in vivo substrate of the chaperone GroEL (Houry et al. 1999). Since oxidation of MetE was found to induce a conformational change, we tested whether GroEL bound preferentially to either form of MetE. GroEL and reduced MetE were incubated together and then injected onto a gel filtration column (Figure 8D). Separate peaks corresponding to GroEL and reduced MetE were observed, indicating that reduced MetE did not associate with GroEL. However, when the glutathionylated protein was incubated with GroEL, only the GroEL peak was observed. The GroEL complex is large enough (approximately 800 kDa) that binding of MetE would not be expected to shift the elution time significantly. Thus, the disappearance of the peak corresponding to oxidized MetE strongly suggests that GroEL specifically binds glutathionylated MetE, leading to coelution.

### The Equilibrium Constant for Glutathionylation of MetE Is Consistent with MetE Oxidation In Vivo

**Figure 9:**
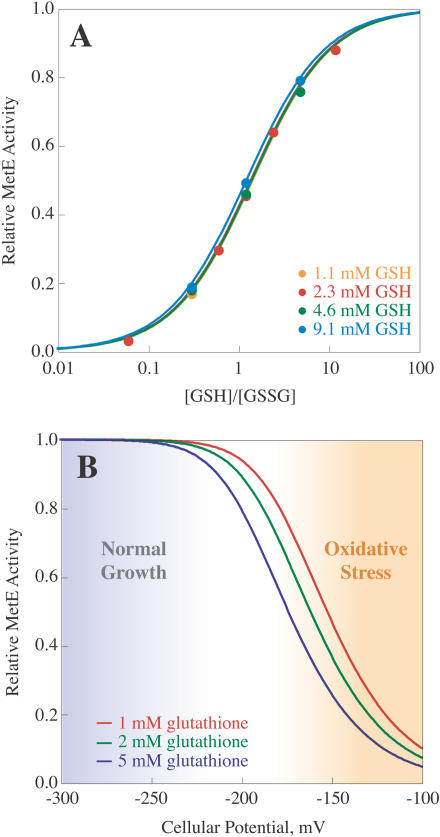
The Equilibrium Constant for MetE Oxidation Is Consistent with MetE Inactivation In Vivo (A) MetE was incubated with the indicated [GSH]/[GSSG] ratios at 1.1 mM (yellow), 2.3 mM (red), 4.6 mM (green), or 9.1 mM (blue) GSH until equilibrium was reached, as judged by a constant level of activity with time. MetE activity was then assayed to determine the relative amount of active reduced enzyme. The equilibrium constant, K_mix_ (see equation 2) was determined to be 1.4 from the plot of relative MetE activity versus [GSH]/[GSSG]. This value was independent of the GSH concentration and dependent only on the [GSH]/[GSSG] ratio, consistent with glutathionylation as the mechanism of inactivation. (B) Cellular redox potentials are often referenced in terms of the GSH–GSSG couple, which depends upon [GSH]^2^/[GSSG]. Since inactivation of MetE occurs via glutathionylation (which is dependent on [GSH]/[GSSG]), a redox potential (which is dependent on [GSH]^2^/[GSSG]) for inactivation of MetE (by equilibrium titration with GSH/GSSG) cannot be determined. In order to provide a physiological context for the K_mix_ determined in vitro, the equilibrium fraction of MetE expected to be active at different glutathione concentrations, 1 mM (red), 2 mM (green), and 5 mM (blue), was determined as a function of the cellular potential as described in the text. The regions designated “normal growth” and “oxidative stress” were inferred from published estimates of typical redox potentials in cells experiencing these conditions (Gilbert 1990).

To gain a better understanding of the thermodynamic sensitivity of MetE to oxidation, we measured the equilibrium constant for formation of the inactive mixed disulfide. MetE was incubated with varied ratios of GSH and GSSG until equilibrium was reached, and then activity was assayed (Figure 9A). The equilibrium constant K_mix_ (equation 2) was determined to be 1.4 and found to be independent of GSH concentration. If formation of an intramolecular disulfide bond were responsible for MetE inactivation, the equilibrium constant should be dependent on the GSH concentration (Gilbert 1995). The independence of the equilibrium constant from GSH concentration provides further support that glutathionylation is the mechanism of inactivation.

In order to estimate the extent that MetE inactivation may be expected in vivo, both the redox potential, which is determined by the [GSH]^2^/[GSSG] ratio, and the total glutathione concentration in the cell ([GSH] + 2[GSSG]) need to be taken into account. Based on the equilibrium constant for GSSG oxidation, MetE activity at equilibrium was calculated for typical cellular glutathione concentrations (1–5 mM [Kosower and Kosower 1978; Åslund et al. 1999]) at various potentials as determined by the GSH–GSSG redox couple (Figure 9B). (At each potential, *E_h_,* the ratio of [GSH]^2^/[GSSG] was calculated from the Nernst equation:



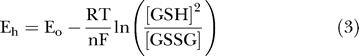



where *E_o_* is –0.252 V at pH 7.2 (using a value of –0.24 V at pH 7.0 with an adjustment of –6.2 mV per 0.1 increase in pH [Schafer and Buettner 2001]), *R* is 8.314 V·C/mol·K, *T* is 310 K, *n* is 2, and *F* is 96,485 C/mol. Based on a total glutathione concentration ([GSH] + 2[GSSG]) of 1, 2, or 5 mM, and the calculated [GSH]^2^/[GSSG], [GSH] and [GSSG] can be determined at each cellular potential. The ratio of [GSH]/[GSSG] may then be used to calculate the relative amount of MetE activity expected at equilibrium by



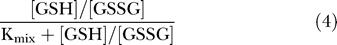



where K_mix_ is 1.4.)

For E. coli growing under normal conditions (–250 mV to –280 mV [Hwang et al. 1992]), more than 97% of the cellular MetE would be expected to be reduced and active. However, in a severely oxidizing environment (greater than –150 mV), inactive MetE may accumulate to an appreciable level. In comparison, Hsp33, which is a redox-regulated chaperone, has a midpoint potential of –170 mV (Jakob et al. 1999). Therefore, thermodynamically, under conditions where Hsp33 is 50% active, MetE would be expected to be 29%–54% oxidized for glutathione concentrations of 1–5 mM. Although GSSG activates Hsp33 via formation of an intramolecular disulfide bond and inactivates MetE by glutathionylation, this suggests that both proteins may have similar sensitivities to oxidation.

However, these calculations assume that the oxidation state of MetE is in equilibrium with GSH and GSSG. Deviations from theoretical values could occur if MetE were oxidized or reduced in vivo by a mechanism other than simple thiol-disulfide exchange. Comparison of the actual amount of oxidized MetE to measurements of the physiological concentrations of GSH and GSSG could therefore provide an indication of the in vivo mechanism of oxidation. Nevertheless, while MetE inactivation by GSSG may be energetically favorable under oxidizing conditions, the apparently slow kinetics make it difficult to conclude whether or not oxidation actually occurs in vivo.

### MetE Is Oxidized In Vivo during Oxidative Stress

**Figure 10:**
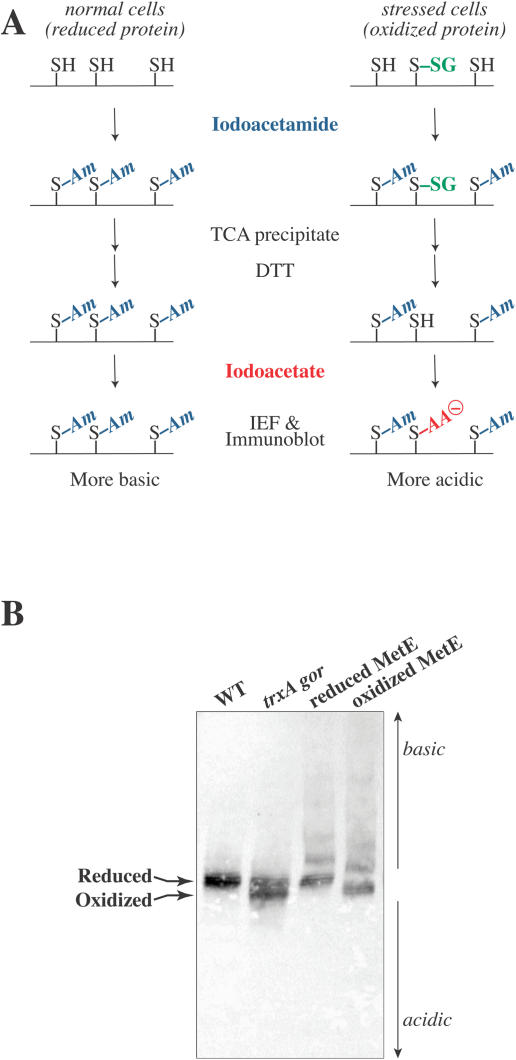
In Vivo Thiol-Trapping Experiments (A) The strategy for in vivo thiol trapping involves brief incubation with iodoacetamide, which is able to efficiently alkylate all available thiols. Oxidized cysteines in the TCA-precipitated proteins are reduced, and the sulfhydryls exposed are then trapped with iodoacetic acid, which adds a negative charge. The two forms of the protein are separated by isoelectric focusing, and MetE is visualized by immunoblotting. Oxidized MetE results in a more acidic band on the gel than reduced MetE. (B) Thiol-trapping experiments were performed on a *trxA gor* strain (WP843) of E. coli (lane 2) as well as the isogenic wild-type strain (DHB4) (lane 1). The position of MetE on the gel may be compared with that of the reduced (lane 3) and GSSG-oxidized (lane 4) purified protein.

Thus, to determine whether our observations are physiologically relevant, we needed to develop a method whereby the oxidation of MetE could be directly observed in vivo. To this end, thiol-trapping experiments were performed as outlined in Figure 10A. Cells growing under normal or oxidative conditions were briefly incubated with iodoacetamide, which is able to quickly penetrate the cell membrane and efficiently alkylate all available reactive thiols. The proteins were precipitated with TCA, and oxidized cysteines were reduced with DTT. The sulfhydryls exposed under these conditions were trapped with iodoacetic acid, which adds a negative charge to the protein for each cysteine trapped. Differently charged forms of the protein were separated by vertical slab isoelectric focusing, and MetE was visualized by immunoblotting. This method of analysis should readily distinguish between glutathionylated MetE (one additional negative charge as compared to reduced MetE) and MetE with an intramolecular disulfide bond (two additional negative charges as compared to reduced MetE). Glutathionylated MetE prepared in vitro is more acidic than fully reduced MetE, as indicated by comparing lanes 4 and 3 in Figure 10B.

In vivo thiol-trapping experiments were performed with logarithmically growing wild-type and *trxA gor* strains of E. coli. Strains mutant in genes for one of the thioredoxins *(trxA)* and glutathione reductase *(gor)* are impaired in their ability to reduce disulfide bonds, resulting in a highly oxidizing cytoplasmic environment (Prinz et al. 1997). Comparison of the thiol status of MetE in a *trxA gor* mutant strain (WP843) with that in the isogenic wild-type strain (DHB4) revealed that approximately 50%–60% of the MetE in the *trxA gor* strain was oxidized in vivo (Figure 10B). Moreover, the shift in the isoelectric point for MetE that was oxidized in vivo is identical to the mobility of glutathionylated MetE prepared in vitro.

**Figure 11:**
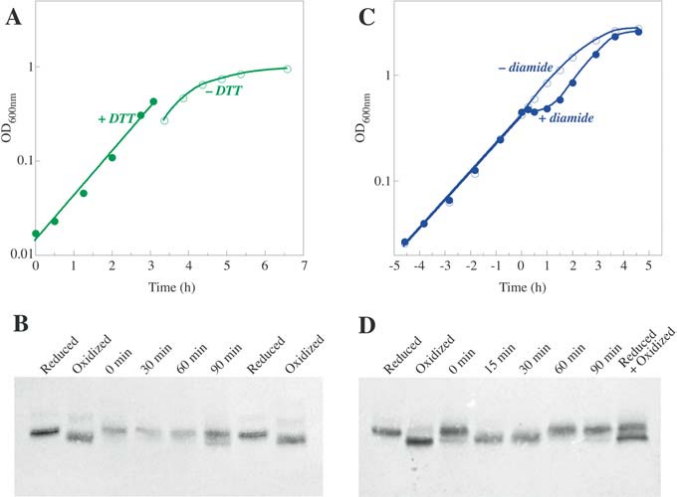
MetE Is Oxidized When E. coli Experience Oxidative Stress (A) Growth of a *trxB gor* strain (WP778) containing a plasmid expressing MetE was monitored by measurement of the OD_600_ during growth in LB medium supplemented with 4 mM DTT (filled symbols). At an OD_600_ of approximately 0.4, cells were filtered to remove the DTT, resuspended in fresh LB medium, and growth was monitored (open circles). (B) In vivo thiol-trapping experiments were performed before and after removal of DTT from the medium. Lanes 1 and 7 contain thiol-trapped samples of purified reduced MetE, and lanes 2 and 8 contain thiol-trapped samples of glutathionylated MetE prepared in vitro by treatment of MetE with GSSG. (C) Growth of wild-type E. coli (strain W3110) in glucose-minimal medium was monitored by the OD_600_ with (filled circles) and without (open circles) the addition of diamide (at OD_600_ approximately 0.5) to a final concentration of 0.9 mM. (D) In vivo thiol-trapping experiments were carried out to assess the oxidation state of MetE at various times following diamide addition. Lanes 1, 2, and 8 contain thiol-trapped samples of purified reduced and/or diamide-oxidized MetE.

E. coli strains containing mutations in *trxB* (which specifies thioredoxin reductase) and *gor* experience more severe disulfide stress than *trxA gor* strains (Prinz et al. 1997). Cells require the presence of DTT in the medium to grow at rates comparable to wild-type strains; growth rapidly halts when DTT is removed. This strain cannot be grown in minimal medium, where wild-type MetE is expressed, so a *trxB gor* strain containing a plasmid specifying MetE (which expresses MetE independently of the cellular methionine concentration) was grown in Luria-Bertani (LB) medium containing DTT. Oxidized MetE was observed within 90 min of DTT removal, while MetE was fully reduced in the presence of DTT (Figure 11A and 11B). Again, the shift in the isoelectric point of MetE oxidized in vivo was indistinguishable from that of glutathionylated MetE prepared in vitro. Together, these experiments provide evidence that MetE is glutathionylated during steady-state growth in two different strains of E. coli that are intrinsically oxidatively stressed.

In order to establish a direct link between oxidative inactivation of MetE and the methionine auxotrophy observed during transient oxidative stresses, we performed thiol-trapping experiments on cells challenged with diamide. When wild-type E. coli cells growing exponentially in glucose-minimal medium were treated with 0.9 mM diamide, MetE was found to be completely oxidized during the lag in growth (Figure 11C and 11D). Identical levels of reduced MetE reappeared within 60 min of the challenge, just prior to the resumption of normal cellular growth. Thus oxidation of MetE is associated with the cellular methionine limitation (described earlier) that is imposed by addition of diamide. Furthermore, these results are consistent with those of Leichert and Jakob (2004), which independently show that MetE is rapidly oxidized in E. coli (wild-type strain DHB4) within 2 min of diamide treatment.

## Discussion

Protein glutathionylation has been increasingly recognized as an important mode of regulation in eukaryotes; however, in *E. coli,* only the activities of OxyR and PAPS reductase have been reported to be modulated by formation of mixed disulfides with glutathione (Kim et al. 2002; Lillig et al. 2003; Potamitou Fernandes and Holmgren 2004). In this study, we have expanded this list by clearly demonstrating that E. coli MetE is also inactivated by glutathionylation in a manner that is stoichiometric and specific for cysteine 645. Moreover, oxidation of MetE was directly observed in vivo concomitant with a methionine auxotrophy induced by oxidative stress.

In general, protein glutathionylation may occur by two different mechanisms. Proteins can undergo thiol-disulfide exchange with GSSG to generate the glutathionylated product (K_mix_ equilibrium in equation 2). Alternatively, either cysteine residues on the protein or the GSH sulfhydryl may be oxidized to a more reactive intermediate, such as a sulfenic acid, followed by formation of the mixed disulfide. In vivo glutathionylation of proteins via thiol-disulfide exchange is dependent on changes in the [GSH]/[GSSG] ratio, while formation of a reactive intermediate would result in glutathionylation independent of the [GSH]/[GSSG] ratio. Alterations in [GSH]/[GSSG] ratios have been associated with increased protein-glutathione mixed disulfides (Chai et al. 1994a); however, there are also reports of extensive glutathionylation occurring in vivo without a measurable change in GSSG concentration or total glutathione levels (Chai et al. 1994a, 1994b). This has been taken as evidence to support a mechanism involving a reactive sulfhydryl intermediate (Thomas et al. 1995).

The ratios of [GSH] to [GSSG] in wild-type (DHB4) and *trxA gor* strains (FÅ378) growing in LB medium were previously reported to be 223 ± 35 and 18 ± 7, respectively (Åslund et al. 1999). Though our experiments were performed with E. coli growing in minimal medium, assuming that the [GSH]/[GSSG] ratios are comparable, more than 99% of the MetE in the wild-type strain (DHB4) should be reduced. Similarly, based on the equilibrium constant for thiol-disulfide exchange with MetE, only approximately 7% of the MetE is expected to be oxidized in the *trxA gor* strain (WP843). Yet 50%–60% of the MetE was found to be oxidized in this strain by thiol-trapping experiments. A [GSH]/[GSSG] ratio of less than 1.4 would be required to accumulate that much oxidized MetE (which corresponds to a redox potential above –174 for 5 mM glutathione and above –152 mV for 1 mM glutathione). Moreover, in vitro oxidation of MetE by GSSG is rather slow, further suggesting that glutathionylation of MetE via thiol-disulfide exchange is not the primary mechanism functioning in vivo. Rather, the in vitro and in vivo data for MetE are consistent with initial formation of a reactive intermediate followed by glutathionylation of cysteine 645, resulting in enzyme inactivation.

Our results are complementary to the observations of Leichert and Jakob (2004) that MetE is one of the proteins most susceptible to oxidation in a *trxA* strain. Since E. coli lacking *trxA* do not experience a substantial amount of cytosolic disulfide bond formation (Prinz et al. 1997), the appearance of oxidized MetE within this environment delineates the extreme sensitivity of the protein to oxidation. The concurrent presence of oxidized peroxide-detoxifying enzymes (alkylhydroperoxide reductase [AhpC] and thioredoxin-linked thiolperoxidase [Tpx]) suggests that endogenously produced reactive oxygen species (e.g., H_2_O_2_) accumulate in the *trxA* strain, since thioredoxin appears to be required to regenerate the reduced thiol status of these proteins. Our preliminary in vitro experiments indicate that glutathionylated MetE is inefficiently reduced by thioredoxin (data not shown), which is not unexpected since glutaredoxins are generally more specific for glutathione-protein adducts (Potamitou Fernandes and Holmgren 2004). Thus, taken together, our study and that of Leichert and Jakob (2004) suggest that the oxidation of MetE observed in *trxA* strains is mediated by elevated reactive oxygen species, perhaps due to an overwhelmed glutaredoxin system. Leichert and Jakob also observed significant oxidation of MetE in wild-type cells treated with H_2_O_2_, further supporting the premise that MetE is acutely vulnerable to reactive oxygen species.

Protein glutathionylation has been suggested to play a protective role in oxidative stress. Studies with purified eukaryotic proteins have shown that incubation with oxidants (e.g., H_2_O_2_) results in irreversible modification, whereas oxidation in the presence of GSH (as would be expected in vivo) yielded reversible glutathionylation (Klatt and Lamas 2000; Hamann et al. 2002; Mallis et al. 2002). Similarly, we found that addition of an excess of H_2_O_2_ to purified MetE appeared to cause irreversible oxidation, presumably because of the formation of sulfinic (–SO_2_) or sulfonic (–SO_3_) acid adducts (data not shown). Thus, glutathionylation of MetE following the formation of a reactive intermediate may serve the dual purpose of modulating enzyme activity while protecting the active site from more extensive damage. Reduction by cellular thioreductases could then readily reactivate the enzyme.

Our data strongly suggest that glutathionylation is reversible in vivo as well as in vitro. Upon the addition of diamide to cells growing in medium lacking methionine, growth is abruptly halted and cells become limited for methionine. In the experiments shown in Figure 11C and 11D, all of the MetE in the cell is oxidized at the 15- and 30-min time points, whereas it is completely reduced after 60 min, just prior to the resumption of growth. Within this time period, the concentration of MetE within the cell does not appear to change, based on the intensity of the band on the immunoblot. In particular, there is no decrease in intensity of the band corresponding to oxidized enzyme between 15 and 30 min, suggesting that MetE is not being degraded. While a lack of change could indicate a balance between new protein synthesis and degradation, we argue that cells that are not growing and are limited for methionine should have a decreased rate of protein synthesis. If new synthesis of MetE were responsible for the reappearance of reduced MetE, all of the oxidized MetE (estimated at 1% of the total protein or 60 μM MetE) must be degraded and new protein synthesized to the same level between 30 and 60 min. Thus, although we cannot definitively rule out the possibility that the reappearance of reduced MetE is due to new protein synthesis, we feel that it is far more likely that MetE is re-reduced once the diamide in the cell is dissipated.

Proteins containing mixed disulfides with glutathione are typically reduced by one of the three glutaredoxin isoforms. The glutaredoxins Grx1 *(grxA)* and Grx3 *(grxC)* are structurally distinct from Grx2 *(grxB),* yet all three proteins are capable of reducing glutathionylated PAPS reductase (Lillig et al. 2003). The levels of Grx2 and Grx3 in E. coli are considerably higher than that of Grx1; however, the three proteins employ different modes of regulation. Grx3 concentrations are relatively constant, whereas OxyR induces Grx1, and levels of Grx2 are growth phase dependent. Grx2 has the highest catalytic activity of the isoforms and has been estimated to contribute up to 80% of the total glutaredoxin activity (Potamitou Fernandes and Holmgren 2004). Since glutaredoxins catalyze the reduction of proteins with high efficiency and specificity, it will be of interest to determine the precise protein or proteins that are responsible for reduction and reactivation of oxidized MetE in vivo.

Based on a recent crystal structure of the Thermatoga maritima MetE homolog, cysteine 645 is located at the entrance to the active site (R. Pejchal and M. Ludwig, personal communication). This places the sulfhydryl in direct contact with the solvent, where it is accessible to oxidation and reduction. Glutathionylation of cysteine 645 at the entrance to the active site may sterically prevent substrate binding, leading to the observed inactivation of the enzyme. Addition of the glutathione adduct also appears to correlate with a weakened binding of the catalytic zinc to the enzyme (data not shown). The active site of MetE is located in a cleft formed between two domains (R. Pejchal and M. Ludwig, personal communication). Addition of the glutathione tripeptide to cysteine 645 may provide enough bulk to push the two domains apart, giving rise to the observed conformational change and increased hydrodynamic radius. CD spectroscopy indicates that oxidation does not result in gross unfolding of MetE (see Figure 8B), yet GroEL was found to bind only to the glutathionylated protein, and not to reduced MetE. Separation of the domains upon addition of glutathione could expose hydrophobic surfaces that allow GroEL to recognize and bind to one of the domains, since the holoenzyme (84.5 kDa) is too large to be accommodated within the cavity formed by the GroEL–GroES complex (Xu et al. 1997). Earlier observations identified MetE as an in vivo substrate for GroEL (Houry et al. 1999); here, we showed that GroEL specifically binds to the glutathionylated protein. GroEL binding may be required to present the enzyme for reduction, as well as to prevent oxidized enzyme from aggregating. Alternatively, binding to GroEL could target the inactive enzyme for eventual degradation.

Oxidant-mediated inactivation of MetE may have broad implications for the cell. Initiation of translation in E. coli requires formylated methionine, and it has been suggested that depletion of methionine and one-carbon pools could block protein translation (Gold 1988). We postulate that the development of methionine auxotrophy via inactivation of MetE may therefore protect stressed cells by slowing the initiation of protein translation. This could safeguard cells from rapid synthesis of peptides under adverse conditions, allowing cellular processes to attend to managing and detoxifying the stress. Interestingly, the conditions that induce a heat shock response in E. coli also affect the translational capacity of the cell, implicating the rate of protein synthesis in triggering of this stress response (VanBogelen and Neidhardt 1990). Studies with rat hepatocytes provide support for a role of protein glutathionylation in the control of protein synthesis under oxidative stress conditions. Upon the addition of *t*-butyl hydroperoxide, rapid inhibition of protein translation was observed, accompanied by an increase in the levels of protein mixed disulfides. It was hypothesized that glutathionylation of key proteins involved in protein synthesis was responsible for the inhibition (Latour et al. 1999). Furthermore, peroxide stress has been found to result in reversible inhibition of protein synthesis in Saccharomyces cerevisiae (Shenton and Grant 2003). This effect was irreversible in cells that lack glutathione, suggesting that protein glutathionylation may protect the cellular translational capacity from irreversible damage. Thus, mechanisms for downregulating the rate of protein translation may be employed by a wide spectrum of organisms experiencing oxidative stress.

The methionine limitation imposed by oxidative stresses could also play a significant role in bacterial quorum sensing. *S*-adenosylmethionine, which is directly formed from methionine, is the precursor to autoinducer AI-2 (see Figure 1) (Chen et al. 2002). A growing body of evidence suggests that AI-2 communicates the metabolic state and growth potential of the cell rather than simply providing a density-dependent signal (DeLisa and Bentley 2002; Xavier and Bassler 2003). Moreover, a shift in the metabolic activity of the cell is postulated to be responsible for modulation of AI-2 signaling in response to a host of environmental stresses (DeLisa et al. 2001a, 2001b). Aerobiosis, H_2_O_2_ treatment, and heat shock were all found to result in decreased AI-2 levels, while addition of DTT or glucose served to increase the accumulation of AI-2 (DeLisa et al. 2001b). Oxidant-mediated inactivation of MetE imposes a methionine limitation, which would then lower the availability of substrate for AI-2 production. Decreased levels of AI-2 in response to methionine-limiting conditions could provide a simple means of communicating the cellular metabolic potential under stress conditions, thereby helping cells to adapt to a non-ideal or even hostile environment.

In conclusion, we have established that MetE, a highly expressed protein in *E. coli,* is inactivated by glutathionylation of cysteine 645 in vitro. Our results, coupled with the findings of Leichert and Jakob (2004), demonstrate oxidation of MetE in E. coli undergoing oxidative stress, as evidenced by high levels of the oxidized protein in three intrinsically stressed strains *(trxA, trxA gor,* and *trxB gor)* as well as wild-type cells challenged with diamide or H_2_O_2_. Our results further show that oxidation of MetE is associated with a methionine limitation imposed by oxidative stress. Hence this study provides insight into a previously unknown, but important aspect of the E. coli cellular response to oxidative stress.

## Materials and Methods

### 

#### Materials.

Electrophoresis-grade urea, Chaps, ampholytes (pH 4–8), ammonium persulfate, TEMED, 10 N sodium hydroxide, and 85% phosophoric acid were obtained from Genomic Solutions (Ann Arbor, Michigan, United States). Iodoacetate and iodoacetamide were purchased from Fluka (Sigma-Aldrich, St. Louis, Missouri, United States). (*6S*)-5-methyltetrahydropteroyltriglutamate was synthesized from pteroyltriglutamate (Schircks Laboratories, Jona, Switzerland), as described previously (Matthews 1986). L-Homocysteine was prepared by hydrolysis of L-homocysteine thiolactone (Drummond et al. 1995). Polyclonal rabbit antibodies to purified wild-type MetE were generated by Lampire Biological Laboratories (Pipersville, Pennsylvania, United States). All other chemicals were obtained from Sigma (St. Louis, Missouri, United States). GroEL was generously provided by Z. Xu (University of Michigan, Ann Arbor, Michigan, United States).

#### Bacterial strains and plasmids.

Wild-type K-12 strain W3110 was obtained from F. C. Neidhardt (University of Michigan, Ann Arbor, Michigan, United States). Wild-type E. coli K-12 strain DHB4 and isogenic E. coli K-12 strains WP843 *(trxA gor)* and WP778 *(trxB gor)* (Prinz et al. 1997) were obtained from U. Jakob (University of Michigan). Overexpression of mutant MetE proteins was performed in E. coli K-12 strain GW2531 *(metE)* (Mulligan et al. 1982). The MetE mutants MetEC>A5 and MetEC>A1–4 were constructed by overlap extension PCR (Ge and Rudolph 1997) using Pfu Turbo (Strategene, La Jolla, California, United States). Plasmid pJG816 (González et al. 1992), specifying wild-type MetE, was used as the template, and the primers used for overlap extension are shown in Table 2. The products were purified by gel electrophoresis and ligated into alkaline phosphatase–treated pJG816. Plasmids containing the mutant genes were isolated after transformation into competent XL1-blue cells by electroporation. Single Cys→Ala mutations of Cys323 (pEM1), Cys353 (pEM2), Cys560 (pEM4), and Cys645 (pEM5) were constructed by overlap extension using pJG816 as the template. For the construction of pEM3 (Cys353Ala, Cys516Ala), pEM2 was used as the template instead of pJG816. For construction of pEM1, pEM2, pEM3, and pEM4, the purified products and pJG816 were digested with BssHII and StuI, while for pEM5, AatII and BssHII were used. MetEC>A1–4 was constructed by subcloning of fragments containing the individual mutations from the appropriate plasmids. Plasmids pEM1 and pEM3 were digested with MluI; the fragment containing the Cys323Ala mutation from pEM1 was ligated into the alkaline phosphatase–treated pEM3 vector to generate pEM6. Similarly, pEM6 was digested with AgeI and BssHII and then ligated into pEM4 that had been digested with the same enzymes to generate pEM7. Plasmids containing pEM5 (MetEC>A5) and pEM7 (MetEC>A1–4) were transformed into E. coli strain GW2531. Mutations were confirmed by DNA sequencing of the entire *metE* gene.

**Table 2:**
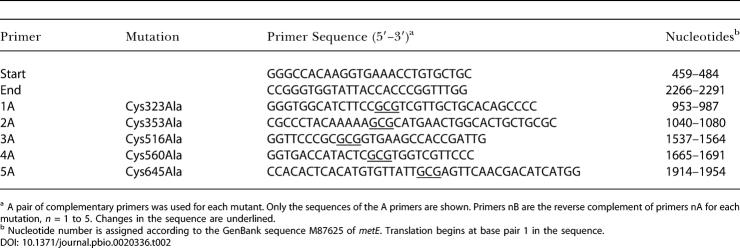
Sequences of Primers Used in the Construction of MetE Mutants

^a^ A pair of complementary primers was used for each mutant. Only the sequences of the A primers are shown. Primers nB are the reverse complement of primers nA for each mutation, *n* = 1 to 5. Changes in the sequence are underlined

^b^ Nucleotide number is assigned according to the GenBank sequence M87625 of *metE*. Translation begins at base pair 1 in the sequence

#### Expression and purification of wild-type and mutant MetE proteins.

Wild-type MetE was expressed using strain GW2531/pJG816, MetEC>A5 was expressed using strain GW2531/pEM5, and MetEC>A1–4 was expressed from strain GW2531/pEM7. Six 1-l portions of LB medium supplemented with 100 μg/ml ampicillin and 0.5 mM zinc sulfate were inoculated with 3 ml of an exponentially growing culture in the same medium (OD_600_ ∼1). Cultures were incubated at 37 °C with shaking at 250 rpm for approximately 30 h before harvesting. The proteins were purified as described previously (González et al. 1992), except that an 800-ml gradient from 100 to 400 mM potassium phosphate (pH 7.2) was used. Purified proteins were dialyzed into 50 mM potassium phosphate buffer (pH 7.2) containing 500 μM DTT, concentrated, and stored at –80 °C.

#### Culture conditions

For examining the effect of oxidative stress in minimal medium, cultures were grown aerobically at 37 °C in a rotary water bath shaker in glucose-minimal morpholinopropane sulfonate (MOPS) medium (Neidhardt et al. 1974) supplemented with 10 μM thiamine. Exponentially growing cultures in the same medium were diluted to an OD_600_ of approximately 0.02–0.04. Where diamide was added to cultures, the concentration of the diamide stock was determined spectrophotometrically (ɛ_296_ = 3,000 M^−1^cm^−1^) (Kosower and Kosower 1995). H_2_O_2_ concentrations were determined similarly (ɛ_240_ = 43.6 M^−1^cm^−1^) (Hildebrandt and Roots 1975; Poosch and Yamazaki 1986).

#### Protein concentration determination and activity assay.

The molar absorption coefficient for MetE at 280 nm was determined to be 157,000 ± 1000 M^−1^cm^−1^ using the Endelhoch method (Pace et al. 1995). The specific activity of MetE was measured using the modified enzyme assay previously described (González et al. 1996), but omitting DTT from the assay mix. Exclusion of DTT did not affect the measured activity. For experiments monitoring enzyme inactivation, aliquots containing 650 pmol of MetE were withdrawn from the oxidation reaction and mixed with activity assay reagents which had been pre-equilibrated to 37 °C. The final assay mixture contained 10 mM potassium phosphate buffer (pH 7.2), 50 mM Tris chloride (pH 7.2), 100 μM magnesium sulfate, 2 mM L-homocysteine, 66 μM *(6S)*-5-methyltetrahydropteroyltriglutamate, and enzyme in a total volume of 400 μl. After a 30-s incubation at 37 °C, the reaction was quenched by addition of 100 μl of 5 N hydrochloric acid/60% formic acid, followed by heating at 88 °C for 10 min. After cooling on ice, the reaction was centrifuged at 14,000*g* for 5 min at 4 °C to remove precipitated protein. The amount of tetrahydrofolate product (converted to methenyltetrahydrofolate by formic acid) was quantitated by its absorbance at 350 nm. Excess GSSG used to oxidize the enzyme did not significantly alter activity during the 30-s assay.

#### Oxidant effects on cysteine oxidation and enzyme activity.

MetE (50 μM) in 100 mM Tris chloride (pH 7.2) was equilibrated to 37 °C, and GSSG was added (final concentration, 5 mM) to initiate the oxidation reaction. The GSSG concentration was established by assay with glutathione reductase (Zander et al. 1998). At time points, aliquots were removed for measurement of cysteine oxidation or enzyme activity (described above). To determine the extent of oxidation, aliquots (100 μl) of the reaction mixture were removed and quenched with 100 μl of ice-cold 20% TCA (w/v). Samples were incubated on ice at least 30 min and then pelleted by centrifugation at 14,000*g* for 30 min at 4 °C. The supernatant was removed, and the pellet was dissolved in 100 mM potassium phosphate buffer (pH 7.3) containing 0.25 mM DTNB, 6 M guanidinium chloride, and 1 mM EDTA. Complete dissolution of the pellet required vortexing for 15–20 min. Reduced cysteines were quantitated by measuring the absorbance at 412 nm after subtraction of a blank containing DTNB and buffer but no protein. The extent of oxidation was determined using the fraction of reduced cysteines at the indicated time compared to the value at time zero (taken as 100%).

#### Preparation of oxidized MetE.

Purified MetE was desalted into 100 mM Tris chloride (pH 7.2) to remove DTT from the storage buffer. The reduced protein (50 μM) was then incubated with 10 mM GSSG for 90 min at 37 °C. Samples were concentrated using Microcon concentrators (Millipore, Billerica, Massachusetts, United States), and GSSG was removed using Bio-Gel P-6 columns (BioRad, Hercules, California, United States) equilibrated with 100 mM Tris chloride (pH 7.2). Reduction and reactivation of GSSG-oxidized MetE was accomplished by incubation of the oxidized enzyme with 2 mM reductant at 37 °C. For preparation of samples of diamide-oxidized MetE to be used as standards for in vivo thiol-trapping assays, diamide (1 mM) was incubated with MetE (10 μM) in 100 mM Tris chloride (pH 7.2) at 37 °C. After 45 min, diamide was removed by gel filtration using Bio-Gel P-6 columns.

#### Fluorescamine assay for glutathionylation of MetE.

Reduced and GSSG-oxidized MetE preparations were exchanged into 50 mM potassium phosphate (pH 7.2) using Bio-Gel P-6 columns. Proteins were then washed three times with the same buffer using Centricon concentrators. MetE (50 μM) was incubated with 2 mM DTT in 50 mM potassium phosphate (pH 7.2) in a volume of 200 μl for 1 h at 37 °C. Each solution was then loaded into a Microcon concentrator and centrifuged at 12,000*g* for 5 min at room temperature. Aliquots (100 μl) of the filtrate were added to 1.7 ml of 200 mM borate buffer (pH 9.0) containing 1 mM *p*-(hydroxymercuri)benzoic acid. While vortexing, 0.6 ml of 1 mg/ml fluorescamine (in acetone) was added. The fluorescence emission of the samples was measured at 485 nm with excitation at 390 nm. A range of GSH concentrations (0–125 mM) was assayed to generate a standard curve. GSH concentrations were determined by DTNB titration (Riddles et al. 1983). The stoichiometry of glutathionylation was calculated by subtracting the results obtained in the absence of DTT from the amount of glutathione released by incubation with DTT and dividing by the enzyme concentration (50 μM). Each form of MetE was assayed in duplicate from two separate preparations.

#### Preparation of disulfide mapping samples.

CDAP dissolved in 200 μl of 100 mM citrate buffer (pH 3) containing 6 M guanidine hydrochloride was added to 100 μl of MetE in 100 mM Tris chloride (pH 7.2) and incubated for 15–20 min at room temperature. The stock concentrations were adjusted so that CDAP was approximately 25-fold in excess over the total thiol concentration. CDAP was removed from the solution by gel filtration, using Bio-Gel P-6 columns equilibrated with 6 M guanidine hydrochloride solution (brought to pH ∼7–8 with potassium hydroxide). Concentrated ammonium hydroxide was added (final concentration 1 M [pH ∼12]), and the reaction was incubated at room temperature for approximately 75 min. Samples were then lyophilized to remove the ammonia and stored at –80 °C. Prior to LC-MS analysis, DTT was added (final concentration was approximately 200 times the concentration of MetE) and allowed to incubate at room temperature for 30 min. Solutions were then diluted into 6 M guanidine hydrochloride solution (adjusted to pH 3 with acetic acid) and characterized by LC-MS.

#### LC-MS analysis.

Samples (250–500 pmol) were injected onto a Vydac (Hesperia, California, United States) 250 mm × 2.1 mm C4 column (214MS52) using an analytical HPLC Surveyor from ThermoFinnigan (San Jose, California, United States). The column was equilibrated with 97% solvent A (water with 0.1% v/v acetic acid and 0.02% v/v TFA) and 3% solvent B (acetonitrile with 0.1% v/v acetic acid and 0.02% v/v TFA), and samples were eluted at a flow rate of 0.25 ml/min with a three-step linear gradient: 3% B for 15 min, 3% to 30% B in 9 min, and 30% to 65% B in 35 min. For LC-MS of the holo-protein, samples were desalted using a two-step linear gradient: 20% B for 10 min, and 20% to 70% B in 25 min. Fractionation was monitored by the UV chromatogram recorded at 280 nm. The eluent was diverted to waste for the first 15 min, and then directly infused into the electrospray ionization source of a ThermoFinnigan LCQ mass spectrometer. Mass spectral data were obtained in the positive mode, and ESI spectra were deconvoluted using the BioMass software provided by ThermoFinnigan.

#### Diamide oxidation of purified MetE.

Oxidation of MetE by diamide was monitored at 37 °C using a Hi-Tech Scientific (Salisbury, United Kingdom) SF-61DX2 stopped-flow spectrophotometer equipped with a xenon light source for single wavelength detection. Diamide (1 mM) and MetE (100 μM) in 100 mM Tris chloride (pH 7.2) were mixed, and reduction of diamide was monitored at 325 nm.

#### Size exclusion chromatography of oxidized and reduced MetE and interaction with GroEL.

MetE (20 pmol) was injected onto an Alltech (Lexington, Kentucky, United States) macrosphere size exclusion HPLC column (250 mm × 4.6 mm) with a 300-Å pore size (7-μm particle size) and run at 0.5 ml/min with 20 mM MOPS (pH 7.5) containing 100 mM potassium chloride and 5 mM magnesium chloride. For GroEL interaction experiments, GroEL (2 μM) was incubated with MetE (1 μM) in 20 mM MOPS (pH 7.5) containing 100 mM potassium chloride and 5 mM magnesium chloride for 30 min at 37 °C before analysis by size exclusion chromatography.

#### CD measurements.

Reduced and oxidized MetE were buffer exchanged into 10 mM potassium phosphate buffer (plus 500 μM DTT for the reduced protein) (pH 7.2). The final filtrates were collected and used as a blank. Samples were centrifuged for 1 h at 14,000*g* at 4 °C and then diluted to 0.4 mg/ml. Far UV CD spectra were recorded using a JASCO (Easton, Maryland, United States) J-810 spectropolarimeter and 0.1 cm cuvettes. Absorbance spectra of the same sample were recorded on a JASCO V-550 UV/Vis spectrophotometer to determine the precise protein concentration. The data were then normalized to a protein concentration of 4.5 μM MetE.

#### Limited tryptic digestion.

Aliquots containing 2.5 mg/ml reduced or oxidized MetE in 50 mM potassium phosphate (pH 7.2) were digested with 0.02% (w/v) trypsin at room temperature. At indicated times, 5-μl samples were quenched with 10 μg of 1-tosylamido-1-lysyl chloromethylketone, and then analyzed by SDS-PAGE (10% gel).

#### Determination of the equilibrium constant for oxidation of MetE by GSSG.

MetE (10 μM) was incubated with varying concentrations of GSH and GSSG in 100 mM Tris chloride (pH 7.2) at 37 °C. GSH (Riddles et al. 1983) and GSSG (Zander et al. 1998) stock concentrations were measured as described. After 5.5 h, aliquots were removed and MetE activity was assayed. Since A_350_ directly correlates to the amount of product in the activity assay, relative MetE activity was calculated by:



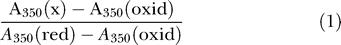



where A_350_(x) is the absorbance at 350 nm for sample x, A_350_(oxid) is for completely oxidized MetE (2 mM GSSG, no GSH), and A_350_(red) is for completely reduced MetE (2 mM GSH, 4 μM [trace] GSSG). The equilibrium constant, K_mix_, was then determined by plotting relative MetE activity versus the [GSH]/[GSSG] ratio.

#### In vivo thiol-trapping experiments.

All E. coli cultures used for in vivo thiol-trapping experiments were grown aerobically at 37 °C. E. coli wild-type (DHB4) and *trxA gor* (WP843) strains were grown in glucose-minimal MOPS medium supplemented with 10 μM thiamine and all amino acids except methionine (Neidhardt et al. 1974), with 0.4 mM arginine used instead of 5.2 mM. Tetracyclin (20 μg/ml) was included in the medium for the *trxA gor* strain. Overnight cultures of exponentially growing cells were diluted to an OD_600_ of approximately 0.02 into the same medium. Cultures were allowed to grow to an OD_600_ of approximately 0.4, and samples were removed for thiol trapping as described below.

For in vivo thiol-trapping experiments performed on the *trxB gor* strain, a plasmid expressing MetE (pJG816) was transformed into electrocompetent *trxB gor* (strain WP778) cells by electroporation. An overnight culture of WP778/pJG816 in LB medium containing 20 μg/ml kanamycin, 50 μg/ml ampicillin, and 4 mM DTT was diluted to an OD_600_ of approximately 0.02 into the same medium (without kanamycin). Care was taken to ensure that an overnight culture from the same inoculum failed to grow when DTT was not included in the medium. The 50-ml culture was grown to an OD_600_ of approximately 0.4, and then filtered into a prewarmed sterile filter flask. The cells were washed twice with prewarmed sterile LB medium to remove residual DTT, and resuspended in 50 ml of prewarmed LB medium. At the indicated time points following DTT removal, samples were taken for alkylation as described.

To assay the oxidation status of MetE in diamide-treated cells, wild-type E. coli were grown in glucose-minimal MOPS medium without methionine as in the growth experiments described above, except that diamide (0.9 mM, final concentration) was added at an OD_600_ of approximately 0.4. Aliquots were removed at intervals following diamide addition for treatment with alkylating agents.

In order to trap oxidized thiols in vivo, 1 ml of culture was added to 250 μl of 0.5 M iodoacetamide in 100 mM Tris chloride (pH 7.2) that had been pre-equilibrated at 37 °C. Samples were incubated 2 min at 37 °C, quenched by the addition of 140 μl of 100% (w/v) ice-cold TCA, and incubated on ice for at least 30 min. Precipitated proteins were pelleted by centrifugation (14,000*g*) for 30 min at 4 °C, washed with cold 10% TCA (once) followed by ethanol (twice), and then dried by incubating approximately 20 min in a 37-°C incubator. The pellet could then be stored at –80 °C prior to secondary alkylation with iodoacetic acid. Pellets were dissolved in 50 μl of alkylation buffer containing DTT (0.1 M Tris chloride [pH 8.7] containing 0.2 M potassium chloride, 1 mM EDTA, 8 M urea, and 10 mM DTT), and incubated 15 min at room temperature. Newly exposed cysteines were trapped by adding 12.5 μl of 0.5 M iodoacetate (104 mg dissolved in 0.5 ml of 1 M potassium hydroxide, followed by addition of 0.5 ml of 1 M Tris chloride [pH 7.3]) and incubating 2 min at room temperature. For some samples, excess DTT was added to quench the reaction; however, this was found to not be necessary. Alkylated proteins were then frozen in liquid nitrogen and stored at –80 °C prior to analysis by isoelectric focusing.

For the reduced and oxidized MetE standards, 25 μl of 0.5 M iodoacetamide was added to 100 μl of 10 μM MetE in 100 mM Tris chloride (pH 7.2) and incubated for 1 min at 37 °C. The reaction was quenched by addition of 125 μl of 20% (w/v) ice-cold TCA and incubation on ice for at least 30 min. The proteins were pelleted by centrifugation (14,000*g*) at 4 °C for 30 min and then washed with cold 10% TCA (once) and either ether (3 times) or 1 M Tris chloride (pH 7.2) followed by water. Pellets were then alkylated with iodoacetate as described for the in vivo culture samples.

#### Vertical slab isoelectric focusing.

The vertical slab isoelectric focusing protocol developed was based on that of Savinova and Jagus (1997) and Robertson et al. (1987). Gels contained 8 M urea, 9.6% glycerol, 3.75% acrylamide, 5% ampholytes, and 1.25% Chaps. Briefly, 6.0 g of urea was dissolved in 2.4 ml of 18 MΩ water, 2.4 ml of 50% glycerol, and 1.56 ml of acrylamide solution (28.36% acrylamide/1.62% bis-acrylamide). 600 μl of ampholytes (pH 4–8) and 780 μl of 20% Chaps were added, and the resulting solution was degassed for approximately 10 min. 25 μl of 10% APS and 20 μl of TEMED were added, and 8 cm × 8 cm × 1.0 cm gels were poured. Gels were allowed to polymerize approximately 30 min and then used immediately. Lanes were carefully washed with cathode buffer.

The sample buffer solution contained 9.5 M urea, 5% chaps, 4.5% glycerol, 0.5 mM lysine–hydrochloride, 2% ampholytes (pH 4–8), 50 mM DTT, and 0.15% SDS. Care was taken not to increase the solution temperature above 30 °C to avoid breaking down urea, which could then carbamylate proteins. Aliquots of the sample buffer were snap-frozen in liquid nitrogen and stored at –20 °C.

An Xcell Surelock Mini-cell gel apparatus from Invitrogen was assembled with the freshly prepared gel, and degassed cathode (upper, 0.1 M sodium hydroxide) and anode (lower, 10 mM phosphoric acid) solutions were added. Samples were diluted at least 10-fold into sample buffer and loaded. Power was applied for 1 h at 100 V followed by 1 h at 200 V and finally 30 min at 500 V. Gels were then stained as described previously (Garfin 1990) or blotted.

#### Immunoblotting.

Gels were electroblotted onto PVDF membrane (Amersham Pharmacia, Piscataway, New Jersey, United States) according to the manufacturer's (Invitrogen) protocol using 1/2X Towbin buffer. Proteins were immunoblotted with a polyclonal rabbit antibody to MetE (1:3,000) and immune complexes were revealed using a fluorescein-linked anti-rabbit antibody (ECF western blotting kit, Amersham Pharmacia) followed by direct detection using a Molecular Dynamics (Sunnyvale, California, United States) Storm phosphorimager.

## Supporting Information

### Accession Numbers

The Swiss-Prot (http://www.ebi.ac.uk/swissprot/) accession numbers for the gene products discussed in this paper are AhpC (P26427), glutaredoxin 1 (P00277), glutaredoxin 2 (P39811), glutaredoxin 3 (P37687), glutathione reductase (P06715), GroEL (P06139), iron superoxide dismutases (P09157), manganese superoxide dismutase (P00448), MetA (P07623), MetE *(C. albicans)* (P82610), MetE *(E. coli)* (P25665), MetE *(T. maritima)* (Q9X112), MetH (P13009), OxyR (P11721), PAPS reductase (P17854), thioredoxin 1 (P00274), thioredoxin reductase (P09625), and Tpx (P37901).

## Abbreviations

AhpC: alkylhydroperoxide reductase
B_12_: cobalamin
CD: circular dichroism
CDAP: 1-cyano-4-dimethylamino-pyridinium tetrafluoroborate
DTNB: dithio-1,4-nitrobenzoic acid
DTT: dithiothreitol
GSH: reduced glutathione
GSSG: oxidized glutathione
H_2_O_2_: hydrogen peroxide
HPLC: high performance liquid chromatography
itz: 2-iminothiazolidine-4-carboxyl
LB: Luria-Bertani
LC-MS: liquid chromatography mass spectrometry
MetA: homoserine transsuccinylase
MetE: cobalamin-independent methionine synthase
MetH: cobalamin-dependent methionine synthase
MOPS: morpholinopropane sulfonate
PAPS: 3′-phosphoadenylylsulfate
TCA: trichloroacetic acid
Tpx: thioredoxin-linked thiolperoxidase
